# EEG-Based Alzheimer’s Disease Recognition Using Robust-PCA and LSTM Recurrent Neural Network

**DOI:** 10.3390/s22103696

**Published:** 2022-05-12

**Authors:** Michele Alessandrini, Giorgio Biagetti, Paolo Crippa, Laura Falaschetti, Simona Luzzi, Claudio Turchetti

**Affiliations:** 1 Department of Information Engineering, Università Politecnica delle Marche, Via Brecce Bianche 12, I-60131 Ancona, Italy; m.alessandrini@univpm.it (M.A.); p.crippa@univpm.it (P.C.); l.falaschetti@univpm.it (L.F.); c.turchetti@univpm.it (C.T.); 2 Clinica Neurologica, Dipartimento di Medicina Sperimentale e Clinica, Università Politecnica delle Marche, Via Conca 71, I-60020 Ancona, Italy; s.luzzi@staff.univpm.it

**Keywords:** recurrent neural network (RNN), deep neural network (DNN), electroencephalography (EEG), Alzheimer’s disease (AD), principal component analysis (PCA), robust PCA (RPCA)

## Abstract

The use of electroencephalography (EEG) has recently grown as a means to diagnose neurodegenerative pathologies such as Alzheimer’s disease (AD). AD recognition can benefit from machine learning methods that, compared with traditional manual diagnosis methods, have higher reliability and improved recognition accuracy, being able to manage large amounts of data. Nevertheless, machine learning methods may exhibit lower accuracies when faced with incomplete, corrupted, or otherwise missing data, so it is important do develop robust pre-processing techniques do deal with incomplete data. The aim of this paper is to develop an automatic classification method that can still work well with EEG data affected by artifacts, as can arise during the collection with, e.g., a wireless system that can lose packets. We show that a recurrent neural network (RNN) can operate successfully even in the case of significantly corrupted data, when it is pre-filtered by the robust principal component analysis (RPCA) algorithm. RPCA was selected because of its stated ability to remove outliers from the signal. To demonstrate this idea, we first develop an RNN which operates on EEG data, properly processed through traditional PCA; then, we use corrupted data as input and process them with RPCA to filter outlier components, showing that even with data corruption causing up to 20% erasures, the RPCA was able to increase the detection accuracy by about 5% with respect to the baseline PCA.

## 1. Introduction

Alzheimer’s disease (AD) is a neurodegenerative disease resulting in cognitive impairments, functional deficits and loss of memory. It is one of the most widespread forms of dementia and has severe effects on all aspects of a patient’s life. Currently no treatments are known for AD and the average survival time is about 4.5 years [1]. The number of subjects affected by AD has been sensibly increasing over the past years and it is expected to reach 15 million by 2050 [2].

AD can be diagnosed at its final stage or in a preclinical, mild stage called mild cognitive impairment (MCI), showing only isolated cognitive deficits that may later result in AD [3,4,5]. Diagnosing AD, especially at early stages, is difficult because symptoms are compatible with normal consequences of ageing or with other pathologies, and because an exact diagnosis requires histological analysis of the brain or other complex exams such as structural magnetic resonance imaging [6].

The use of electroencephalography (EEG) has recently grown as a means to diagnose neurodegenerative pathologies such as AD [7,8,9,10,11,12,13]. EEG is a non-invasive test that records the electrical activity of the brain measured at different sites on patient’s scalp, resulting in indirect information about the physiological conditions of the brain. The resulting waveforms can be divided in different frequency bands, which in turn can be ascribed to different cerebral activities.

Several studies have shown that variations in frequency patterns of EEG signals can be related to a variety of neurodegenerative diseases, including AD and MCI [14,15,16,17,18,19]. However, analyzing EEG data can be a difficult task, because these exams typically generate large amounts of data, which must be precisely scanned for the specific pattern one is looking for. If done manually, this must be performed by trained medical professionals.

That is why a considerable amount of research exists in the application of machine learning techniques to automatically detect interesting patterns in EEG signals, in order to diagnose a specific disease. This can be treated as a pattern recognition problem and can take advantage of the continuous improvements of deep learning techniques. Existing algorithms can be categorized in two main groups: (i) statistical machine learning techniques, (ii) deep learning-based techniques.

In the first category various machine learning methods, such as *k*-nearest neighbors (*k*-NN) and support-vector machines (SVM), have been adopted [11,13,20,21,22]. The second category, i.e., deep learning-based techniques, includes deep neural networks (DNNs) and in particular recurrent neural networks (RNNs) [23,24,25,26,27,28,29,30]. The latter are especially suited for time-based data series, such as EEG.

Regarding the deep learning-based techniques, in [13] authors propose two EEG features, namely, epoch-based entropy (a measure of signal complexity) and bump modeling (a measure of synchrony) and demonstrate that these features are sufficient for efficient discrimination between subjective cognitive impairment (SCI) patients, MCI patients, possible AD patients, and patients with other pathologies, obtaining a classification accuracy of 81.8% to 88.8%. In [20], a novel analytical framework combining fuzzy learning and complex network approaches has been proposed for the identification of AD with multichannel scalp-recorded EEG signals, obtaining a highest accuracy of 97.12%. In [23,24] authors propose a strategy to use RNN that can handle missing data, which is common in healthcare data. Missing data are considered as arising due to various reasons, such as the nature of data collection procedures, subjects’ dropping out of studies, or mistakes in data collection, so that entire examination results are missing, but these papers do not specifically handle the case of missing data segments within a signal trace as can happen if the measurement device has a wireless link. In [23], a multiclass area under the operating curve (mAUC) of 0.86 and a balanced class accuracy (BCA) of 0.79 was obtained, while in [24] a prediction performance of clinical diagnosis drop from a BCA of 0.935 in year 1 to a BCA of 0.810 in year 6 was achieved.

Special attention was also devoted to the extraction of features from the EEG signal, as shown in [31,32], where the authors propose a framework for the accurate identification of different motor-imagery (MI) tasks in brain-computer interface (BCI), exploring different feature extraction methods and obtaining good results with the application of the multiscale principal component analysis (MSPCA) method. The same aim, that is, increasing the classification outcome of different MI signals by selection of efficient features, was pursued in [33], applying the EEG preprocessing to several neural networks while a custom CNN was implemented in [34].

In the context of feature extraction, robust principal component analysis (RPCA) [35] is a method widely applied when the data observations are subject to sensible degradation. Thus, this method can be useful for the treatment of the EEG signal that can be affected by artifacts that can arise during the collection with, e.g., a wireless system that can lose packets.

The aim of this paper is primarily to validate the effectiveness of RPCA applied to corrupted EEG signals, in order to clear the signals and then extract features through PCA. Once the features are extracted, a custom RNN is implemented in order to classify patients as healthy or suffering from AD, training the network with a dataset obtained from EEG recordings of 35 hospitalized subjects belonging to both categories. In doing that we operate at three different levels of data elaboration, showing that performance of the network is progressively increased:

- Untransformed EEG data (time series);
- Features extracted from EEG data through principal component analysis (PCA);
- Corrupted signals, after filtering them with different algorithms, namely, robust PCA (RPCA) and multiscale PCA (MSPCA).

Specifically, the latter test showed that applying RPCA in a suitable way, compatible with the data representation, leads to better results in the case of corrupted input data with respect to other filtering algorithms, or no filtering at all.

The rest of the paper is organized as follows. In Section 2 we summarize the basic principles of RNNs. Section 3 describes the dataset adopted in the experiments. Section 4 describes the data processing applied to improve the RNN performance, including PCA and RPCA transformations. Section 5 reports the details of the proposed RNN architecture and its main features, together with a description of the hardware and software that was used. Experimental results are presented in Section 6 and discussed in Section 7. Finally, some conclusions are drawn in Section 8.

## 2. Brief of RNNs

In this work, we make use of RNNs with a structure similar to that employed in our previous work [36]; in this section, we thus only report a summary of their architecture and operational principles for easier reference.

**Figure 1:**
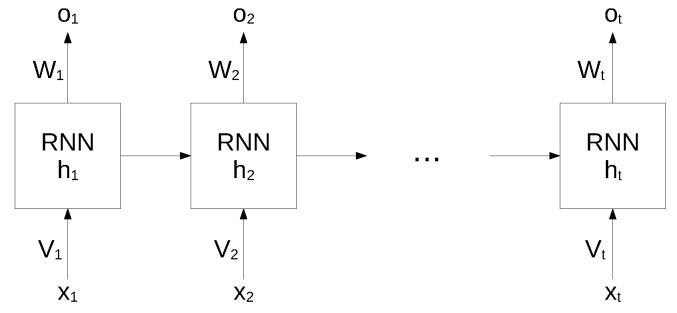
Structure of an RNN. Reproduced under CC-BY from [36].

While standard neural networks are characterized by a complete interconnection between adjacent layers, recurrent neural networks can map target vectors from the full history, as represented by the previous map. The structure of an RNN network is shown in Figure 1.

In this architecture, each node maintains a current hidden state $h_{t}$ and produces an output $o_{t}$ by using the current input $x_{t}$ and the previous hidden state $h_{t-1}$ as follows:

(1) $$\begin{array}{c} h_{t}=f\left(W_{h}h_{t-1}+V_{h}x_{t}+b_{h}\right) \end{array}$$

(2) $$\begin{array}{c} o_{t}=f\left(W_{o}h_{t}+b_{o}\right) \end{array}$$

where *W* and *V* are the weights of the hidden layers in recurrent connections, *b* is the bias for hidden and output states and *f* is an activation function.

**Figure 2:**
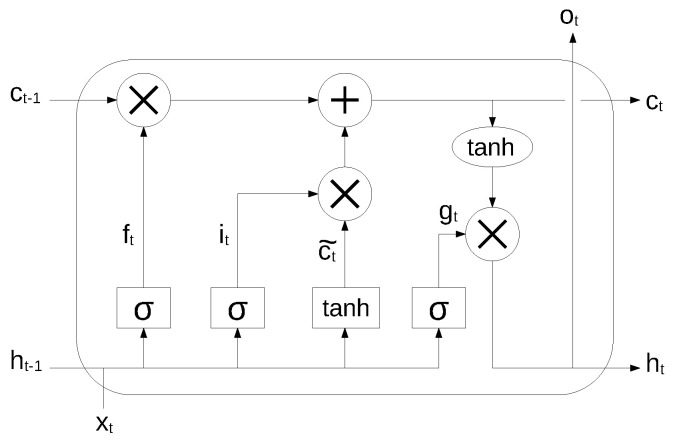
LSTM cell unit. Reproduced under CC-BY from [36].

An RNN is very effective in modeling the dynamics of a continuous data sequence, but it may suffer from the problem of gradient disappearance and explosion [37] if modeling long sequences. In order to solve this problem, Hochreiter et al. [38] proposed a variant of RNN, based on the long short-term memory (LSTM) cell unit, which combines learning with model training, with no additional domain knowledge. The structure of the LSTM unit is shown in Figure 2.

The long-term state, short-term state and the output of each layer at each time step are described by the following equations:

(3) $$\begin{array}{c} f_{t}=\sigma \left(W_{x_{f}}^{T}\;x_{t}+W_{h_{f}}^{T}\;h_{t-1}+b_{f}\right) \end{array}$$

(4) $$\begin{array}{c} i_{t}=\sigma \left(W_{x_{i}}^{T}\;x_{t}+W_{h_{i}}^{T}\;h_{t-1}+b_{i}\right) \end{array}$$

(5) $$\begin{array}{c} \tilde{c_{t}}=\tanh\left(W_{x_{c}}^{T}\;x_{t}+W_{h_{c}}^{T}\;h_{t-1}+b_{c}\right) \end{array}$$

(6) $$\begin{array}{c} g_{t}=\sigma \left(W_{x_{g}}^{T}\;x_{t}+W_{h_{g}}^{T}\;h_{t-1}+b_{g}\right) \end{array}$$

(7) $$\begin{array}{c} c_{t}=f_{t}⊗c_{t-1}+i_{t}⊗\tilde{c_{t}} \end{array}$$

(8) $$\begin{array}{c} o_{t}=h_{t}=g_{t}⊗\tanh\left(c_{t}\right) \end{array}$$

where $W_{x_{f}}$, $W_{x_{i}}$, $W_{x_{c}}$, $W_{x_{g}}$ are the weight matrices associated to the matching connected input vector, $W_{h_{f}}$, $W_{h_{i}}$, $W_{h_{c}}$, $W_{h_{g}}$ are the weight matrices for the short-term state from the previous time step, and $b_{f}$, $b_{i}$, $b_{c}$, $b_{g}$ are bias terms. The symbol ⊗ means point-wise multiplication.

## 3. Dataset

We used a dataset obtained from EEG recordings of 35 hospitalized subjects, 20 of which suffered from Alzheimer’s disease, and are denoted in the following as “AD”, while 15 were healthy, indicated with “N” (Normal). The data were obtained from the subjects as part of ordinary medical diagnostic procedures performed in a hospital environment; all the data were collected according to the Declaration of Helsinki, had been properly anonymised, and informed consent was obtained at the time of original data collection.

EEG data are obtained with electrodes placed in several points along the subject’s scalp, each recording a signal from a specific brain region. The specific equipment used to acquire the dataset is a Galileo BE Plus PRO Portable, Light version, providing 37 total inputs, of which 22 unipolar and 8 bipolar AC/DC inputs. The electrodes were applied in the standard 10–20 system (referring to distances between adjacent electrodes, expressed in percentage of the total available space on the skull).

Table 1 shows a summary of the consistency of the dataset, expressed in terms of the total duration of the recordings for the two classes of subjects. To perform our experiments, in the following the dataset will be split in 30 subjects for training and 5 for testing (see Section 6 for details).

The demographic distribution of the involved subjects was chosen so to be as similar as possible in the two groups. The AD patients‘ mean age is 74.16 years (SD 4.64), their mean education is 7.55 years (SD 3.50) and the mean illness duration is 2.4 years (SD 0.64; range 1–3 years). The healthy subjects’ mean age is 73.61 years (SD 5.81) and their mean education is 8.07 years (SD 3.92). The demographic variable do not differ in the two groups: age (t=0.29, p=0.77), education (t=0.38, p=0.70), sex ($\chi^{2}=0.40$, d.f.=2, p=0.48).

The dataset is composed of files in EDF format (European data format), a standard file format typically used for exchange and storage of medical data. Each recording includes a slightly different set of signals, all sampled at 128 Hz. All the signals for a specific subject have been acquired synchronously, so that all traces have the same duration, but of course the duration is different for different subjects. Data traces are organized in rows, with the number of different signals spanning the first dimension (rows, indeed) and time samples spanning the second dimension (columns).

## 4. Data Processing

**Figure 3:**
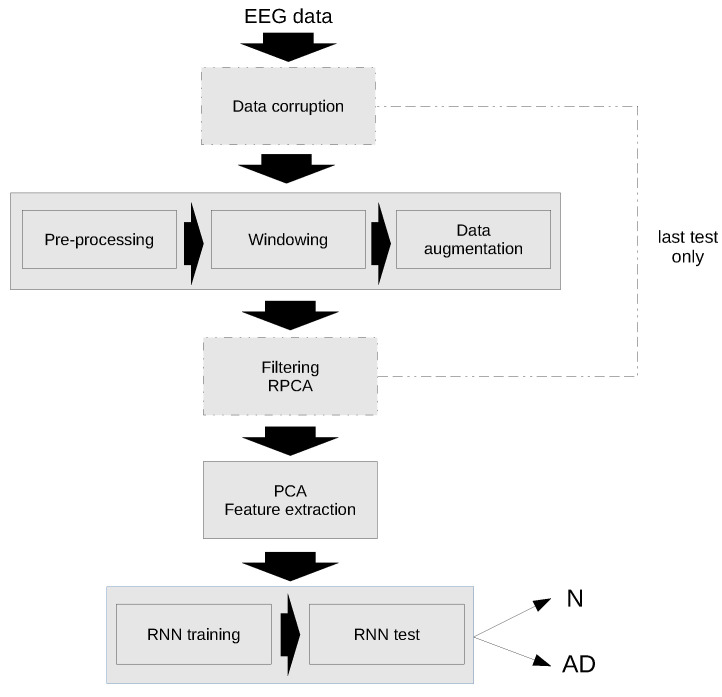
Flow chart of the proposed algorithm for Alzheimer’s disease Recognition.

A schematic diagram of the classification algorithm is shown in Figure 3. As previously mentioned, the first test has been performed with the original data, while in the second test the data have been corrupted and an additional filtering step has been applied using RPCA.

The individual steps of the algorithm are explained in detail in the following sections.

### 4.1. Data Pre-Processing

Before using the dataset as input for the following steps, some preliminary pre-processing was required. First of all, the set of signals included in every subject is slightly different, varying from 21 to 23 signals from the following list: Fp1, Fp2, Fpz, EKG, F7, F3, Fz, F4, F8, C3, Cz, C4, T3, T4, T5, T6, P3, Pz, P4, O1, O2, I, II, MK. Moreover, they are in different order for every subject.

For that reason, in order to have a coherent set of input data, we isolated the subset of common signals for all the subjects, resulting in a set of 16 signals, namely: Fp1, Fp2, F7, F3, F4, F8, T3, C3, C4, T4, T5, P3, P4, T6, O1, O2, and extracted and reordered them. In the process, data have been saved in “npy” format (Python NumPy numerical format) to be more conveniently processed in the following steps. Moreover, the matrices have been transposed, so as to have the time variable on the first dimension, as required by the RNN toolkit used.

It follows from the previous steps that every subject’s data result in a matrix of dimension n×16, where *n* is the number of time points. It can be seen that a maximum of 7 tracks have been discarded from every subject. The resulting matrices have no associated information about the meaning of the specific tracks anymore, but they are used as 16-dimensional temporal series associated to every subject.

A further cleaning of the data has been performed for the presence, in some of the recordings, of artificial signals (square waves) at the beginning and at the end of actual EEG data. Those artificial signals are used to test or synchronize the equipment before starting acquiring real data from the electrodes. Leading and trailing intervals have been removed when needed to discard such spurious data. The duration of those intervals is indeed negligible with respect to the total duration.

Finally, since different signals exhibit significative differences in magnitude, statistical standardization has been applied to all the signals (columns), that is, data were scaled so that the resulting mean and standard deviation were 0 and 1, according to the formula

(9) $$y\left[n\right]=\frac{x[n]-\mu }{\sigma }$$

with μ and σ being the original mean and standard deviation, respectively. This was experimentally proven to largely improve the final classification accuracy, especially when using PCA, which selects principal components based on the variance of the input data.

### 4.2. Data Windowing

In order to be processed by the RNN, input data are split along the time axis in windows of fixed size, possibly overlapping by a given amount. The window length and overlapping are important hyper-parameters in neural networks, as well as in many other machine learning algorithms [39,40]. Being *w* the number of samples in a window and *o* the number of overlapping samples between adjacent windows, the nth data window corresponds to samples in the range

(10) $$\left[n(w-o),n(w-o)+w-1\right]$$

with n>=0.

The resulting input matrix for the RNN has therefore dimension N×w×16, where *N* is the number of resulting data windows and *w* is the window length.

To find the best combination for our particular network, we did a series of tests with various values of the two parameters (Section 6).

### 4.3. Data Augmentation

Since the number of inputs belonging to the two different classes are not equally represented, the network might end up being biased towards a specific class. A simple technique to address this problem is oversampling [41], a form of data augmentation where the data from classes with less occurrences are duplicated as needed, so that the data used for training are more uniformly distributed among the different classes.

Jittering with gaussian noise has been applied to the duplicated data, so not to have identical data in the training set.

Oversampling has been applied before each training phase to the subset of subjects used for training, leaving the test subjects unaltered. Table 2 shows an example of data distribution before and after data augmentation, for windows of 512 samples with 25% overlap, considering the subset of 30 training subjects.

### 4.4. Principal Component Analysis

A first set of tests has been performed using the original uncorrupted data, both in the time domain (no feature extraction, letting the RNN itself adapt to the raw input data) and applying PCA to the input data matrix. PCA is commonly used in a vast range of applications in order to reduce data dimensionality while simultaneously improving the signal-to-noise ratio of the resulting data. Consequently, dimensionality reduction not only simplifies the computation, but also generally improves the final accuracy, isolating the most relevant features of the signal.

PCA-based data reduction of a matrix can be performed by first factorizing the original matrix through singular value decomposition (SVD). Given a generic matrix X of size m×n and assuming m>n, SVD can factorize it as:

(11) $$X=USV^{T}$$

where S (n×n) is a diagonal matrix containing the singular values, and U, V have dimension m×n, n×n, respectively, and contain the singular vectors.

Dimensionality reduction can then be achieved by truncating S to the first *p* singular values (p<n), provided they are sorted in descending order, and consequently truncating V to the first *p* columns (let the new matrix be called $V_{p}$). This decomposition ensures the preservation of the largest part of the variance of the original matrix for a given rank *p*.

A new matrix can then be substituted to X:

(12) $$X^{*}=XV_{p}\;\left(m\times p\right)$$

which contains the principal components of X and has lower rank.

The choice of the *p* parameter must take into account the relative magnitude of singular values and can be chosen according to different criteria (see below for a possible choice).

A problem arising in the case of neural networks is that the input data matrix, once the original data are split into windows, is actually a 3-dimensional tensor, with size (in this particular case) N×w×16, where *N* is the number of data windows and *w* is the window length, 16 being the number of selected EEG signals. As a concrete example, for the case with data windows of size 512 and 25% overlapping, using data from the 30 training subjects, the input tensor has size 16,198×512×16.

Calling A the original input tensor, a strategy must therefore be devised to reshape A into a 2-dimensional matrix, in order to apply SVD and PCA.

A first strategy might consist in converting the original data to size N×(16w), that is flattening every data window to a single one-dimensional vector:

(13) $$A\in R^{N\times w\times 16}\to A^{\prime }\in R^{N\times (16w)}$$

However, experiments showed this to not be the best strategy, since the number of input features (*N*) is comparable to the dimension of the features themselves, leading to a poor statistical representation. In the previous example, indeed, the size of $A^{\prime }$ would be 16,198 × 8192.

A strategy that proved to perform better is instead the following: first we define a new input matrix $A^{\prime }$ having size N×16×w (obtained by transposing data windows); then, it is converted to size (16N)×w (flattening the first two dimensions):

(14) $$A\in R^{N\times w\times 16}\to A^{\prime }\in R^{N\times 16\times w}\to A^{\prime \prime }\in R^{(16N)\times w}$$

This results in a matrix $A^{\prime \prime }$ with the first dimension much greater than the second one, as desired, and as assumed in (11). In the previous example, $A^{\prime \prime }$ has size 259,168 × 512. At this point we can apply SVD and PCA reduction as previously defined to $A^{\prime \prime }$ and compute $V_{p}$ of size w×p. We can then compute the principal components of $A^{\prime \prime }$ by multiplying it by $V_{p}$, defining:

(15) $$A^{\prime \prime \prime }=A^{\prime \prime }V_{p}\;\left(\left(16N\right)\times p\right)$$

This new matrix can then be reverted to a 3-dimensional tensor (N×16×p) and finally, in order to have the 16 signal tracks correctly appearing on the last dimension, we can transpose the second and third dimensions again, to obtain the final tensor suitable to be used as an input the RNN application:

(16) $$A^{\prime \prime \prime }\in R^{(16N)\times p}\to A^{*}\in R^{N\times p\times 16}$$

**Figure 4:**
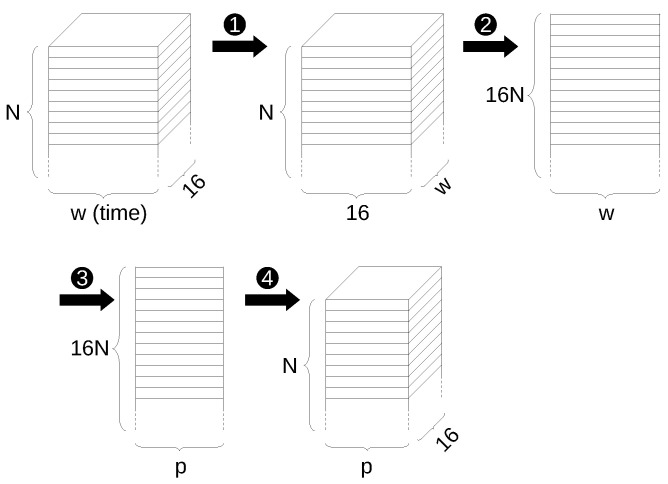
Steps to apply PCA reduction to 3-D input tensor: ➊ transposing second and third dimension, ➋ flattening first two dimensions, ➌ dimension reduction through PCA, ➍ converting back to original form.

The previous steps of reducing the input data to a 2-dimensional matrix for application of the PCA and converting it back to a 3-dimensional tensor are shown schematically in Figure 4.

**Figure 5:**
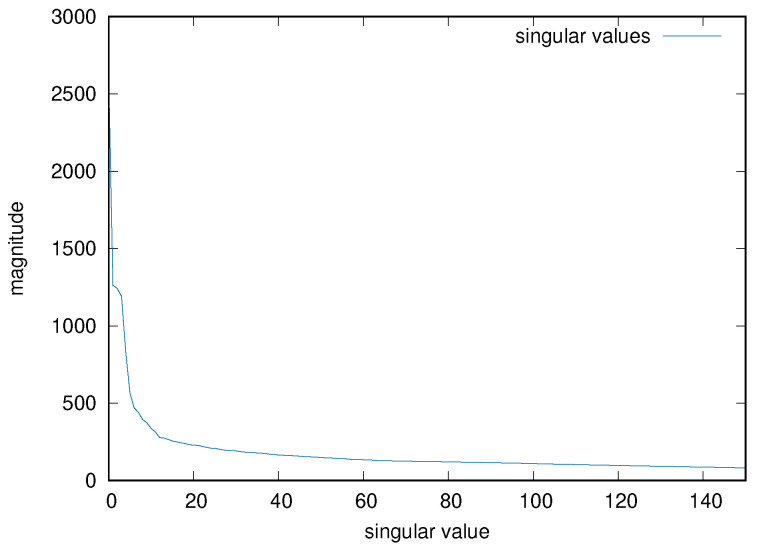
Singular value magnitude of an example input matrix, limited to the first 150 values.

As mentioned, one must choose the parameter *p* for the truncation of the number of principal components, as an hyper-parameter of the resulting system. An empirical value of 50 has been chosen for all the input matrices, after inspecting some of the results of the PCA decomposition for several input cases. Figure 5 shows, for example, the magnitude of the first 150 singular values for the input matrix used in the previous examples. In the previous example, therefore, the final input tensor $A^{*}$ has size 16,198 × 50 × 16.

### 4.5. Robust PCA

Robust PCA is an enhancement to the traditional PCA decomposition, that can be applied when the matrix contains corrupted observations, even for sensible levels of corruption [35].

Given a (possibly large) data matrix M and assuming that it can be decomposed as

(17) $$M=L_{0}+S_{0}$$

where $L_{0}$ is a low-rank matrix and $S_{0}$ is sparse, RPCA offers a method to recover $L_{0}$ and $S_{0}$ accurately and efficiently, even without prior information on their dimension and magnitude.

RPCA is widely used when the data observations are subject to sensible degradation; in many cases the low-rank $L_{0}$ component models the original data, distributed in a low-dimensional subspace, while the sparse $S_{0}$ matrix represents the corruption components (outliers). Typical applications are image recognition and classification, signal filtering, user ranking and many others [35,42,43,44,45].

The RPCA decomposition is performed with an iterative algorithm, estimating $L_{0}$ and $S_{0}$ within a desired tolerance. Details are shown in Algorithm 1. In the algorithm, M is the input matrix, $L_{k}$ and $S_{k}$ are the results as computed at the *k*th iteration;

   $S_{\tau }:R\to R$ is a shrinkage operator such that

(18) $$S_{\tau }\left(x\right)=\mathrm{sign}\left(x\right)\;\max\left(|x|-\tau ,0\right)$$

extended to matrices by applying it to each element;

   $D_{\tau }$ is a threshold operator such that

(19) $$D_{\tau }\left(X\right)=US_{\tau }\left(\Sigma \right)V^{T}$$

where $X=U\Sigma V^{T}$ (singular value decomposition);

μ and λ, finally, are parameters that must be chosen according to the nature of the data.

In this work, we apply the RPCA to compensate for degradation in the original EEG signals, and show that it can improve the RNN performance in those cases. Operatively, we apply RPCA to the matrix obtained in (14), that is the matrix obtained by reducing the dimension of the original 3-dimensional tensor input; subsequently, dimensional reduction by PCA is applied to the $L_{0}$ matrix as computed by the RPCA.
**Algorithm 1** RPCA algorithm**Input:** 
M
1:**procedure** Compute RPCA2:    $S_{0}=Y_{0}=0$3:    **repeat**4:        Compute $L_{k}=D_{1/\mu }\left(M-S_{k-1}+\mu^{-1}Y_{k-1}\right)$5:        Compute $S_{k}=S_{\lambda /\mu }\left(M-L_{k}+\mu^{-1}Y_{k-1}\right)$6:        Compute $Y_{k}=Y_{k-1}+\mu \left(M-L_{k}-S_{k}\right)$7:    **until** $∥M-L_{k}-S_{k}∥$ < tolerance8:**end procedure**
**Output:** 
$L_{k}$, $S_{k}$                           ▹*k* = last computed step


## 5. RNN Architecture

**Figure 6:**
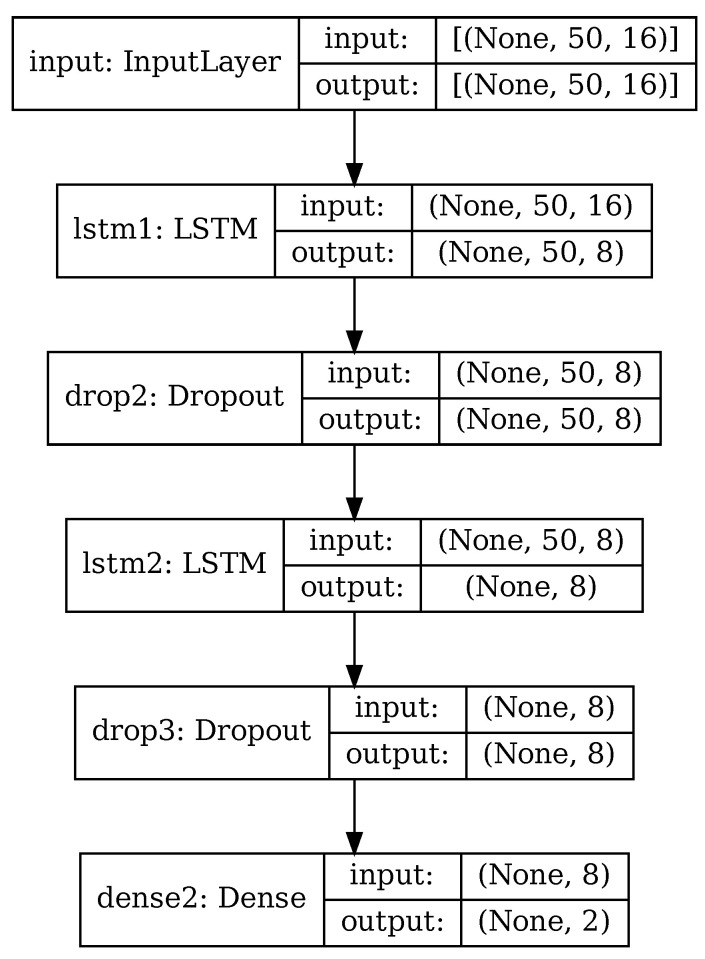
RNN architecture.

The RNN used in this paper is depicted in Figure 6, for the case of PCA applied and 50 principal components retained. It is based on architectures commonly used with time-based sensor data [36,40,41,46] and consisting of a mix of LSTM cells and fully connected layers.

Input data consist in a series of m×n matrices, depending on whether PCA reduction is used or not. When using original data with no PCA, input data are time series of size w×16, with *w* being the size of data windows as described in Section 4.2. When using PCA or RPCA, input data are the principal components extracted from the original data, of size p×16 as in (16).

The core of the recurrent neural network consists of two cascaded LSTM layers, whose internal architecture was briefly explained in Section 2. Each LSTM layer is followed by a dropout layer that randomly discards some of the input data. All the intermediate layers have size 8; this hyper-parameter has been chosen experimentally, starting with a larger value and decreasing it until the network accuracy varied significantly.

Finally, there is a fully-connected layer (dense) of size 2. In this layer the generic nth neuron produces an output $y_{n}$ depending on the $x_{1},\ldots x_{m}$ inputs to the layer and the $w_{nj}$ neuron weights associated to every input, specifically:

(20) $$y_{n}=\phi \left(\sum_{j=1}^{m}w_{nj}x_{j}+b_{n}\right)$$

where ϕ is an activation function and $b_{n}$ is a bias value.

The fully-connected layer performs the classification in one of the 2 classes, according to the sparse categorical crossentropy loss function assigned to the network. The loss function, or cost function in general terms of an optimization problem, represents the error to be minimized by the training process. The specific formula for the error according to the categorical crossentropy function is

(21) $$J\left(w\right)=-\frac{1}{N}\sum_{i=1}^{N}\left[y_{i}\log\left(\hat{y_{i}}\right)+\left(1-y_{i}\right)\log\left(1-\hat{y_{i}}\right)\right]$$

where *w* is the set of model parameters, e.g. the weights of the RNN, *N* is the number of input test features, $y_{i}$ and $\hat{y_{i}}$ are the actual and predicted classes, respectively, expressed numerically.

One of the problems to be aware of when designing a neural network is overfitting, that is, the generated model might be performing well on the training dataset, but poorly on new, unseen data. We employed a combination of several techniques to reduce overfitting: use of dropout layers, as just mentioned, to discard random portions of data; data augmentation with random noise applied (Section 4.3); randomly mixing input data from training and validation subjects (Section 6) so to have representative samples from all the subjects for both training and validation.

As an example, Table 3 shows the details of the individual layers in the case where PCA has been performed and 50 principal components retained (p=50→m,n=50,16), matching the configuration of Figure 6. The RNN in this configuration has 1394 trainable parameters.

### Hardware and Software

The RNN was developed with TensorFlow 2.6.1 and Keras 2.6.0; the rest of the computations were performed using Python NumPy and SciPy suites, unless otherwise specified in Section 6. For the purpose of design and hyper-parameter optimization, the network and the related algorithms were initially developed on the Google Colaboratory platform; the final computations were then performed on a computer with an Intel Core i7-6800K CPU, 32 GiB of RAM and an NVIDIA GeForce GTX 1080 GPU.

All the software developed for this article is publicly available at https://github.com/MAlessandrini-Univpm/rnn-eeg-ad, published 14 April 2022.

## 6. Experimental Results

As mentioned, the first set of tests has been performed using both original temporal data and principal components extracted through PCA. Every test has been repeated for different combinations of window length and overlapping, to find the best results.

It is common practice, when training a neural network, to split the dataset in subjects used for training and subjects for independent test, and to further split the training data in two sets: data actually used to fit the network weights, and validation data to monitor the performance of the network during the various training epochs.

Since the number of different subjects in the dataset is not large, and different subjects inevitably have substantial differences in their data, the statistical distribution of the data might not be uniform enough, and so choosing a single partition for training and validation might not lead to representative results.

So we decided to split the training input data in two sets for training and validation, respectively, with a ratio of 75%/25%, after shuffling the input data windows, so to better statistically integrate data from different training subjects. The subjects used for test are isolated from the beginning and not participating in the training/validation phase. The exact procedure is showed in Algorithm 2.
**Algorithm 2** Testing algorithm1:**procedure** Compute testing accuracies2:    $S_{1}$ = set of training subjects3:    $S_{2}$ = set of testing subjects4:    **for all** cases No-PCA / PCA **do**5:        **for all** combinations of window and overlap **do**6:           Train RNN with subjects $S_{1}$, splitting in training + validation7:           Test trained network on subjects $S_{2}$8:        **end for**9:    **end for**10:**end procedure**

The RNNs were trained with the following parameters:

- 5 different window sizes ranging from 1 to 5 s;
- window overlapping of 0, 25% and 50%;
- data augmentation (Section 4.3);
- 50 principal components (when PCA is used);
- 30 subjects used from training and validation, 5 for testing;
- 20 training epochs.

Table 4 and Table 5 show the test results when using original temporal data and when applying PCA, respectively. It can be seen that using PCA results in sensibly higher accuracy (97.9% versus 79.3% for the best cases), as it is expected for the benefits of PCA on this class of data.

**Figure 7:**
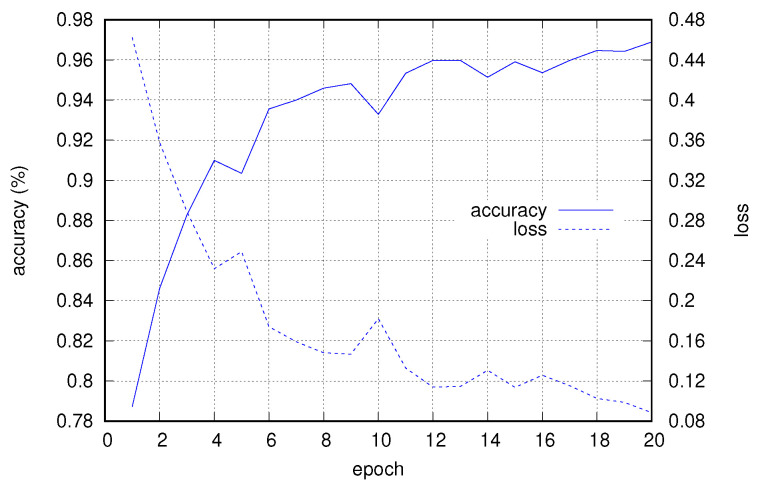
Accuracy and loss progress for validation data with respect to training epochs.

Figure 7 shows the progress of accuracy and loss (computed on the validation subset) with respect to the training epochs, for the case with a window of size 512 and 25% overlap.

**Figure 8:**
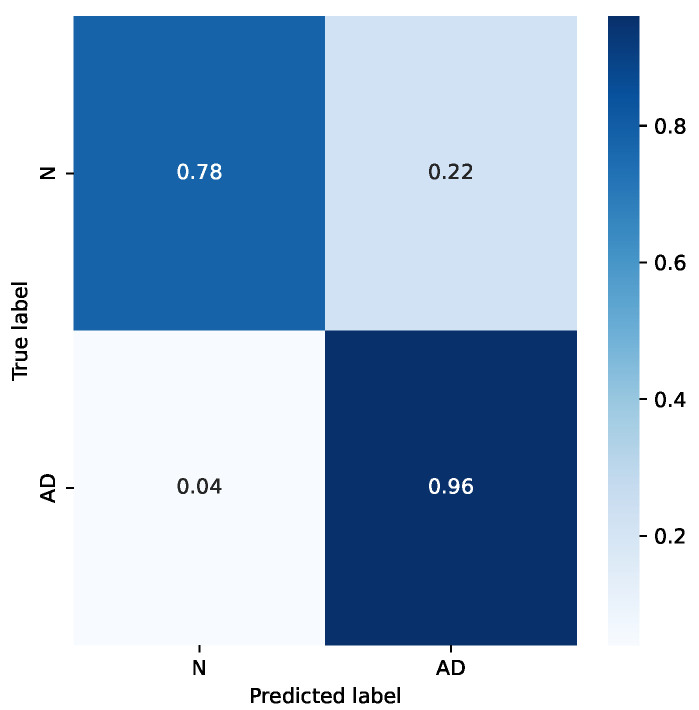
Confusion matrix for a sample case.

It is interesting to analyze the difference in accuracy for the two classes. Figure 8 shows the confusion matrix for the mentioned example. It can be seen that the accuracy for the “Normal” class is sensibly worse. This is likely due to the smaller number of subjects in that category, and also for the choice of subjects in the dataset: subjects in the “Normal” category come from hospitalized patients, too, who either were treated for other diseases or belong to risk groups.

### Test of Robustness with RPCA

To test the robustness of the RNN with respect to corruption of original signals, we simulated a situation where some of the data are lost, for example due to bad connections of the electrodes, limited capacity of the elaboration system with respect to the data bandwidth, or packets lost in a hypothetical wireless connection between the electrodes and the acquisition system.

**Figure 9:**
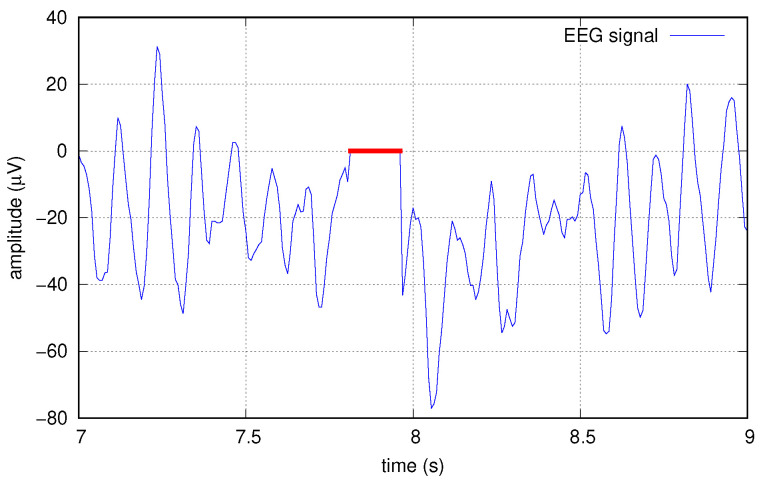
Example of data corrupted with a hole (red).

To this end, we artificially corrupted the data at several increasing levels by creating “holes” in the original signals, before any other elaborations; such holes are sequences of zeros, applied simultaneously to all the 16 tracks. Holes are applied at random times with a probability *p* chosen in the set 1/1000,1/500,1/200,1/100. Single holes have a random length, too, with a normal distribution of mean length 20, resulting in average erasure rates of 2%, 4%, 10%, and 20%, respectively. Figure 9 shows an example of a hole created in one of the signals.

The corrupted signals are filtered with RPCA, as explained in Section 4.5, before the actual PCA and dimension reduction. As a matter of comparison, another test has been done using MSPCA as a filtering technique.

MSPCA [47] combines the ability of PCA to extract the crosscorrelation or relationship between the variables, with that of orthonormal wavelets to separate deterministic features from stochastic processes, and approximately decorrelate the autocorrelation among the measurements. In MSPCA, several PCA decompositions are performed at different resolution levels, keeping a different number of components at every level, according to a suitable algorithm. Every decomposition level can highlight different signal components, such as noise.

MSPCA has shown to be an effective method for noise removal in automated detection systems, possibly combined with other dimension reduction techniques, such as empirical wavelet transform or multivariate variational mode decomposition [31,32,33,34].

In this article, we used the Matlab MSPCA implementation [48], running on Matlab R2022a. As in the RPCA, we apply MSPCA to the matrix obtained in (14), then dimensional reduction by PCA is applied to the matrix obtained by the MSPCA.

For RPCA, a fundamental aspect is choosing the right parameters, especially the μ parameter (Section 4.5). For every value of *p* (probability of data corruption) several tests have been performed to find the optimal value of μ. In real applications, this parameter must be evaluated according to the statistical properties of the analyzed signal and noise.

For MSPCA, the best results were obtained experimentally as follows. Decomposition is performed at 5 levels, using the Daubechies least-asymmetric wavelet with 4 vanishing moments (sym4). A first run is performed to compute the retained principal components according to the Kaiser’s rule. Then a second run is performed after removing the principal components of level 1, in order to filter out the signal noise. See [48] for implementation details.

**Figure 10:**
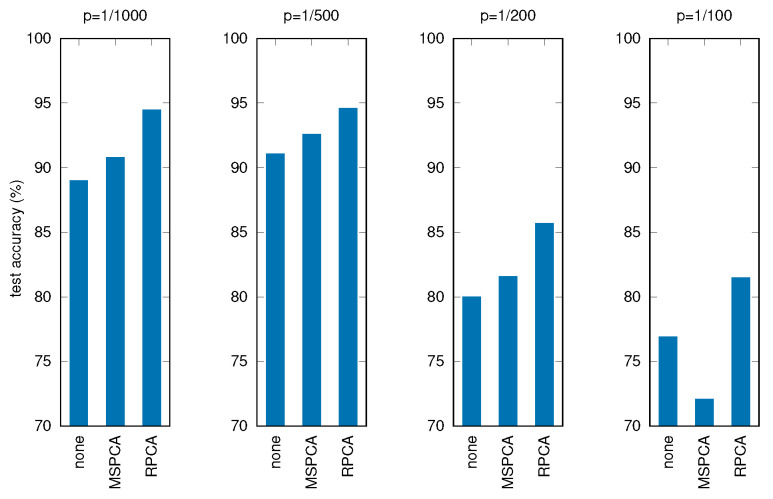
Test accuracy for corrupted signals with different filtering techniques (*p* is the corruption probability).

Table 6 and Figure 10 show the results of these tests, where all the other parameters and procedures are the same as in the previous experiments, comparing the three cases: no filtering applied before PCA, filtering through MSPCA, filtering through RPCA.

It can be seen that applying RPCA always results in better final accuracies, holding good performances for the RNN even with severely corrupted input data.

## 7. Discussion

The performed experiments have shown that the designed RNN can recognize subjects with AD, from the dataset used for the tests, with a good accuracy (97.9%), under optimal choice of hyper-parameters and after computing the principal components of input data with PCA (as opposed to untransformed time-based data). These results confirm the effectiveness of DNNs, and specifically RNNs, for time-based data series, in finding significant patterns in classes of data with no previous knowledge of the underlying system.

Moreover, the last experiment has shown that for corrupted data, specifically with bursts of missing information, applying RPCA as a filtering stage can maintain a good accuracy, even in the case of high corruption rate. This is due to the property of RPCA of removing outlier components from the main signals, filtering out noise and other artifacts with a different statistical distribution with respect to the relevant information.

It is interesting to compare the results with the other studies mentioned in the introduction (Section 1). Starting with methods based on RNNs, Refs. [23,24] address the problem of missing data, even if not in the form of whole data segments as we do, and reach a classification accuracy of 79% and 93.5%, respectively, the latter decreasing to 81% on a longer timescale. Refs. [27,28] combine RNNs with wavelet preprocessing, obtaining an accuracy under 90%. Even if the data were processed in different ways, such as explicitly adding noise in our case, it can be seen that our results are consistent with the cited ones, while also addressing the possible problem of signal degradation.

The same considerations hold when comparing to methods based on strictly statistical machine learning, such as *k*-NN and SVM, especially with regard to the added robustness to corrupted data. Ref. [11] analyzes data in the frequency domain, using the fast Fourier transform (FFT), in order to link variations in spectral components to AD; three alternative classification algorithms are then compared, namely, SVM, decision trees and rule-based classifiers, in the end obtaining a prediction accuracy up to 90%. Ref. [13] extracts two specific features from EEG data— epoch-based entropy (a measure of signal complexity) and bump modeling (a measure of synchrony)—and, together with orthogonal forward regression (OFR) and SVM algorithms, shows an accuracy of 91.6%. In our previous work [22] we also used RPCA at the feature extraction phase, before applying several machine learning algorithms, like *k*-NN, decision tree, SVM, naïve Bayes, and obtained a maximum accuracy of 93.18% with SVM. This proves that RPCA is useful as a pre-processing stage for EEG signals since it improves recognition rates in both traditional machine learning algorithm and, as the current paper shows, with deep learning methods.

Finally, the accuracy obtained by our algorithm is comparable to more expensive and complex techniques, such as magnetic resonance imaging (MRI), of which a comprehensive review of its application to machine learning for AD diagnosis can be found in [49].

## 8. Conclusions

In this paper, an RNN is built to classify medical subjects as healthy or suffering from Alzheimer’s disease, using EEG data obtained by a sample of subjects from both categories. Several elaborations are applied to the original data to obtain a better accuracy, in particular principal component analysis, that results in an accuracy of more than 97% on the test data, using optimal hyper-parameters. Most importantly, it is shown that using corrupted input data and applying RPCA to them, a good accuracy can be maintained, and a consistent improvement of around 5% with respect to baseline PCA, can be achieved even when the corruption rate is sensibly high, with up to 20% erasures in the input samples.

As a possible future evolution of the proposed algorithms, their application could be extended to the classification of multiple neurodegenerative pathologies besides AD, for which data collection is already in progress, or to the identification of contributions of the different brain regions to the various patologies.

## Figures and Tables

**Table 1:** Dataset details.

Class	Subjects	Duration (s)
N	15	17,932
AD	20	28,586
Total	35	46,518

**Table 2:** Number of data windows before and after oversampling (example for windows of 512 samples with 25% overlap).

Class	Original	Oversampled
N	5424	8099
AD	8099	8099
Total	13,523	16,198

**Table 3:** Details of RNN layers.

Layer	Input Size	Output Size	Parameters
LSTM 1	(-, 50, 16)	(-, 50, 8)	832
Dropout 1	(-, 50, 8)	(-, 50, 8)	0
LSTM 2	(-, 50, 8)	(-, 8)	544
Dropout 2	(-, 8)	(-, 8)	0
Dense	(-, 8)	(-, 2)	18

**Table 4:** Experimental results for original temporal data, best result is displayed in bold.

window samples	128			256			384			512			640		
window duration (s)	1			2			3			4			5		
window overlap (%)	0	25	50	0	25	50	0	25	50	0	25	50	0	25	50
input features	36,493	48,642	72,961	18,240	24,310	36,468	12,157	16,198	24,294	9115	12,148	18,214	7290	9711	14,562
training time (s)	399.3	527.7	787.5	288.5	402.8	603.4	270.7	359.7	539.9	250.3	334.3	500.6	236.7	315.7	472.3
test time (s)	1.3	1.9	2.8	1.6	1.4	2.1	1.0	1.2	1.9	0.9	1.2	1.8	0.9	1.2	1.7
test accuracy (%)	56.7	77.5	**79.3**	51.9	45.2	42.3	50.7	68.6	57.3	56.9	51.3	48.1	59.5	67.5	52.4

**Table 5:** Experimental results for PCA data using 50 principal components, best result is displayed in bold.

window samples	128			256			384			512			640		
window duration (s)	1			2			3			4			5		
window overlap (%)	0	25	50	0	25	50	0	25	50	0	25	50	0	25	50
input features	36,493	48,642	72,961	18,240	24,310	36,468	12,157	16,198	24,294	9115	12,148	18,214	7290	9711	14,562
training time (s)	267.5	354.4	531.1	124.8	179.3	279.5	91.7	120.3	179.4	69.3	90.9	135.1	55.6	73.5	109.2
test time (s)	0.5	1.2	1.7	1.0	0.3	0.7	0.2	0.2	0.4	0.2	0.2	0.6	0.1	0.2	0.2
test accuracy (%)	62.4	73.6	87.0	92.8	96.0	**97.9**	96.9	90.5	90.1	94.6	88.4	95.6	93.4	96.3	93.2

**Table 6:** Test accuracy for corrupted signals with different filtering techniques (*p* is the corruption probability, data loss represent the average number of missing samples).

*p* (‰)	Data Loss (%)	No Filter (%)	MSPCA (%)	RPCA (%)
1	2	89.0	90.8	94.5
2	4	91.1	92.6	94.6
5	10	80.0	81.6	85.7
10	20	76.9	72.1	81.5

## Data Availability

The data presented in this study are available on request from the corresponding author.

## References

[1] Xie J., Brayne C., Matthews F.E. (2008). Survival times in people with dementia: Analysis from population based cohort study with 14 year follow-up. BMJ.

[2] Jeong J. (2004). EEG dynamics in patients with Alzheimer’s disease. Clin. Neurophysiol..

[3] Petersen R.C. (2009). Early diagnosis of Alzheimer’s disease: Is MCI too late?. Curr. Alzheimer Res..

[4] Petersen R.C. (2004). Mild cognitive impairment as a diagnostic entity. J. Intern. Med..

[5] Gauthier S., Reisberg B., Zaudig M., Petersen R.C., Ritchie K., Broich K., Belleville S., Brodaty H., Bennett D., Chertkow H. (2006). Mild cognitive impairment. Lancet.

[6] Biagetti G., Crippa P., Falaschetti L., Luzzi S., Santarelli R., Turchetti C. (2019). Classification of Alzheimer’s disease from structural magnetic resonance imaging using particle-Bernstein polynomials algorithm. Smart Innov. Syst. Technol..

[7] Tsolaki A., Kazis D., Kompatsiaris I., Kosmidou V., Tsolaki M. (2014). Electroencephalogram and Alzheimer’s disease: Clinical and research approaches. Int. J. Alzheimer’s Dis..

[8] Kulkarni N.N., Bairagi V.K. Electroencephalogram based diagnosis of Alzheimer Disease. Proceedings of the 2015 IEEE 9th International Conference on Intelligent Systems and Control (ISCO).

[9] Falk T.H., Fraga F.J., Trambaiolli L., Anghinah R. (2012). EEG amplitude modulation analysis for semi-automated diagnosis of Alzheimer’s disease. EURASIP J. Adv. Signal Process..

[10] Dauwels J., Vialatte F., Cichocki A. (2010). Diagnosis of Alzheimer’s disease from EEG signals: Where are we standing?. Curr. Alzheimer Res..

[11] Fiscon G., Weitschek E., Felici G., Bertolazzi P., De Salvo S., Bramanti P., De Cola M.C. Alzheimer’s disease patients classification through EEG signals processing. Proceedings of the 2014 IEEE Symposium on Computational Intelligence and Data Mining (CIDM).

[12] Lehmann C., Koenig T., Jelic V., Prichep L., John R.E., Wahlund L.O., Dodge Y., Dierks T. (2007). Application and comparison of classification algorithms for recognition of Alzheimer’s disease in electrical brain activity (EEG). J. Neurosci. Methods.

[13] Houmani N., Vialatte F., Gallego-Jutglà E., Dreyfus G., Nguyen-Michel V.H., Mariani J., Kinugawa K. (2018). Diagnosis of Alzheimer’s disease with Electroencephalography in a differential framework. PLoS ONE.

[14] Coben L.A., Danziger W.L., Berg L. (1983). Frequency analysis of the resting awake EEG in mild senile dementia of Alzheimer type. Electroencephalogr. Clin. Neurophysiol..

[15] Arenas A.M., Brenner R.P., Reynolds C.F. (1986). Temporal slowing in the elderly revisited. Am. J. EEG Technol..

[16] Cibils D. (2002). Dementia and qEEG (Alzheimer’s disease). Supplements to Clinical Neurophysiology.

[17] Kowalski J.W., Gawel M., Pfeffer A., Barcikowska M. (2001). The diagnostic value of EEG in Alzheimer disease: Correlation with the severity of mental impairment. J. Clin. Neurophysiol..

[18] Besthorn C., Förstl H., Geiger-Kabisch C., Sattel H., Gasser T., Schreiter-Gasser U. (1994). EEG coherence in Alzheimer disease. Electroencephalogr. Clin. Neurophysiol..

[19] Locatelli T., Cursi M., Liberati D., Franceschi M., Comi G. (1998). EEG coherence in Alzheimer’s disease. Electroencephalogr. Clin. Neurophysiol..

[20] Yu H., Zhu L., Cai L., Wang J., Liu J., Wang R., Zhang Z. (2020). Identification of Alzheimer’s EEG With a WVG Network-Based Fuzzy Learning Approach. Front. Neurosci..

[21] Tanveer M., Richhariya B., Khan R., Rashid A., Khanna P., Prasad M., Lin C. (2020). Machine learning techniques for the diagnosis of Alzheimer’s disease: A review. ACM Trans. Multimed. Comput. Commun. Appl. (TOMM).

[22] Biagetti G., Crippa P., Falaschetti L., Luzzi S., Turchetti C. (2021). Classification of Alzheimer’s disease from EEG signal using robust-PCA feature extraction. Procedia Comput. Sci..

[23] Nguyen M., Sun N., Alexander D.C., Feng J., Yeo B.T. Modeling Alzheimer’s disease progression using deep recurrent neural networks. Proceedings of the 2018 International Workshop on Pattern Recognition in Neuroimaging (PRNI).

[24] Nguyen M., He T., An L., Alexander D.C., Feng J., Yeo B.T., Initiative A.D.N. (2020). Predicting Alzheimer’s disease progression using deep recurrent neural networks. NeuroImage.

[25] Gong S., Xing K., Cichocki A., Li J. (2020). Deep Learning in EEG: Advance of the Last Ten-Year Critical Period. arXiv.

[26] Li G., Lee C.H., Jung J.J., Youn Y.C., Camacho D. (2020). Deep learning for EEG data analytics: A survey. Concurr. Comput. Pract. Exp..

[27] Petrosian A., Prokhorov D., Lajara-Nanson W., Schiffer R. (2001). Recurrent neural network-based approach for early recognition of Alzheimer’s disease in EEG. Clin. Neurophysiol..

[28] Petrosian A.A., Prokhorov D., Schiffer R.B. Early recognition of Alzheimer’s disease in EEG using recurrent neural network and wavelet transform. Proceedings of the Wavelet Applications in Signal and Image Processing VIII.

[29] Wang T., Qiu R.G., Yu M. (2018). Predictive modeling of the progression of Alzheimer’s disease with recurrent neural networks. Sci. Rep..

[30] Yang S., Chen H.C., Wu C.H., Wu M.N., Yang C.H. (2021). Forecasting of the prevalence of dementia using the lstm neural network in Taiwan. Mathematics.

[31] Sadiq M.T., Yu X., Yuan Z., Aziz M.Z. (2020). Motor imagery BCI classification based on novel two-dimensional modelling in empirical wavelet transform. Electron. Lett.

[32] Sadiq M.T., Yu X., Yuan Z., Aziz M.Z., ur Rehman N., Ding W., Xiao G. (2022). Motor Imagery BCI Classification Based on Multivariate Variational Mode Decomposition. IEEE Trans. Emerg. Top. Comput. Intell..

[33] Sadiq M.T., Yu X., Yuan Z. (2021). Exploiting dimensionality reduction and neural network techniques for the development of expert brain–computer interfaces. Expert Syst. Appl..

[34] Sadiq M.T., Aziz M.Z., Almogren A., Yousaf A., Siuly S., Rehman A.U. (2022). Exploiting pretrained CNN models for the development of an EEG-based robust BCI framework. Comput. Biol. Med..

[35] Candès E.J., Li X., Ma Y., Wright J. (2011). Robust principal component analysis?. J. ACM (JACM).

[36] Alessandrini M., Biagetti G., Crippa P., Falaschetti L., Turchetti C. (2021). Recurrent Neural Network for Human Activity Recognition in Embedded Systems Using PPG and Accelerometer Data. Electronics.

[37] Hochreiter S. (1998). The vanishing gradient problem during learning recurrent neural nets and problem solutions. Int. J. Uncertain. Fuzziness Knowl. Based Syst..

[38] Hochreiter S., Schmidhuber J. (1997). Long short-term memory. Neural Comput..

[39] Biagetti G., Crippa P., Falaschetti L., Focante E., Martínez Madrid N., Seepold R. (2020). Machine Learning and Data Fusion Techniques Applied to Physical Activity Classification Using Photoplethysmographic and Accelerometric Signals. Procedia Comput. Sci..

[40] Musci M., De Martini D., Blago N., Facchinetti T., Piastra M. (2020). Online Fall Detection using Recurrent Neural Networks on Smart Wearable Devices. IEEE Trans. Emerg. Top. Comput..

[41] Eddins S. (2018). Classify ECG Signals Using LSTM Networks. https://blogs.mathworks.com/deep-learning/2018/08/06/classify-ecg-signals-using-lstm-networks/.

[42] Hubert M., Engelen S. (2004). Robust PCA and classification in biosciences. Bioinformatics.

[43] Rahmani M., Li P. Outlier detection and robust PCA using a convex measure of innovation. Proceedings of the Advances in Neural Information Processing Systems 32 (NeurIPS 2019).

[44] Su J., Tao H., Tao M., Wang L., Xie J. (2017). Narrow-band interference suppression via RPCA-based signal separation in time–frequency domain. IEEE J. Sel. Top. Appl. Earth Obs. Remote Sens..

[45] Xu H., Caramanis C., Mannor S. (2012). Outlier-robust PCA: The high-dimensional case. IEEE Trans. Inf. Theory.

[46] Chevalier G. (2016). LSTMs for Human Activity Recognition. https://github.com/guillaume-chevalier/LSTM-Human-Activity-Recognition.

[47] Bakshi B.R. (1998). Multiscale PCA with application to multivariate statistical process monitoring. AIChE J..

[48] MathWorks (2022). wmspca—Multiscale Principal Component Analysis. https://it.mathworks.com/help/wavelet/ref/wmspca.html.

[49] Lazli L., Boukadoum M., Mohamed O.A. (2020). A Survey on Computer-Aided Diagnosis of Brain Disorders through MRI Based on Machine Learning and Data Mining Methodologies with an Emphasis on Alzheimer Disease Diagnosis and the Contribution of the Multimodal Fusion. Appl. Sci..

